# Utilization of recurrent laryngeal nerve monitoring during thyroid surgery in China: a point prevalence survey (2015–2023)

**DOI:** 10.1097/JS9.0000000000002084

**Published:** 2024-09-06

**Authors:** Yishen Zhao, Peiyao Wang, Gianlorenzo Dionigi, Jiedong Kou, Changlin Li, Fang Li, Tie Wang, Wen Tian, Kewei Jiang, Ping Wang, Hao Zhang, Hui Sun

**Affiliations:** a Department of Thyroid Surgery, China-Japan Union Hospital of Jilin University, Jilin Provincial Key Laboratory of Translational Medicine in Surgery, Jilin Provincial Engineering,Laboratory of Thyroid Disease Prevention and Treatment, Changchun; b Department of Thyroid & Hernia Surgery, Medical Department of General Surgery, Chinese People’s Liberation Army General Hospital, Beijing; c Department of Gastroenterological Surgery, Peking University People ‘s Hospital, China; d Department of Thyroid Surgery, The Second Affiliated Hospital of Zhejiang University School of Medicine, Hangzhou; e Department of Thyroid Surgery, The First Hospital of China Medical University, Shenyang, China; f Division of Surgery, Istituto Auxologico Italiano IRCCS (Istituto di Ricovero e Cura a Carattere Scientifico); g Department of Pathophysiology and Transplantation, University of Milan, Milan, Italy

**Keywords:** experience sharing, intraoperative neuromonitoring, questionnaires, thyroid surgery, training

## Abstract

**Background:**

The survey aimed to elucidate the complete range of national practices, including all technical and non-technical aspects, as well as surgical stratification and maturation, of the use of intraoperative neuromonitoring (IONM) during thyroid surgery in China.

**Materials and methods:**

Six national questionnaires, developed by the Chinese Neural Monitoring Study Group (CNMSG) between 2015 and 2023, were used to collect and analyze data regarding the clinical application, education, and scientific research related to IONM in Chinese medical institutions.

**Results:**

Among the surveyed hospitals, 45% reported an average annual surgical volume exceeding 3000 cases, with 82.5% performing more than 80% of the surgeries for malignant thyroid tumors. Additionally, 97.5% of the hospitals reported a less than 3% incidence of postoperative hoarseness with IONM. Statistical analysis from 2011 to 2015 found that the incidence of postoperative hoarseness decreased by 30% in 2013 compared with 2011, when the technology was introduced. Preoperative and postoperative laryngoscopies were routinely performed by 82.5% and 15% of the hospitals, respectively. For 65% of the hospitals, the publication of the Chinese edition of neuromonitoring guidelines in 2013 prompted the utilization of IONM technology. An average annual number of IONM applications exceeding 500 cases (18.5% the average volume) was reported by 80% of the hospitals, while 62.5% reported a cumulative number of applications greater than 5000 cases (47.1% the average cumulative volume). Regarding technical parameters, 75% of the hospitals reported an intraoperative V1 amplitude of greater than 500 µV, and 70% reported an intraoperative loss of signal (LOS) rate of less than 3%. 92.5% of the surveyed hospitals believed that IONM could help identify dissociated nerves, and 95% of the surveyed hospitals believed that IONM could reduce nerve damage. However, 72.5% of the respondents thought that cost was the main limitation. Furthermore, 67.5% of the hospitals reported that half of their thyroid surgical team members were trained in IONM, with 17.5% reporting that all team members were trained. Areas for reinforced training included IONM research methods and directions (72.5%) and analysis and treatment of abnormal EMG signals (72.5%). Research projects related to IONM were conducted by 42.5% of the hospitals, while 52.5% had published papers on neuromonitoring.

**Conclusions:**

IONM was independently and incrementally associated with the annual surgical volume. This survey emphasized the importance of national collaboration and/or a registry for the uptake, consolidation, and development of CNMSG consensus.

## Introduction

Highlights

- Six national surveys by CNMSG revealed widespread use of IONM in China, with 45% of hospitals performing more than 3000 thyroid surgeries annually.
- IONM implementation led to a significant drop in postoperative hoarseness, with 97.5% of hospitals reporting an incidence of less than 3%.
- Preoperative laryngoscopy was standard in 82.5% of hospitals; however, only 15% performed routine postoperative examinations.
- Guidelines publication in 2013 influenced 65% of hospitals to adopt IONM, improving surgical outcomes and team training.
- Financial constraints were cited as the primary barrier to IONM adoption despite its proven benefits in nerve identification and damage reduction.

The greatest advantage of the intraoperative neuromonitoring (IONM) technique is that it assists in protecting the nerves and reduces the risk of nerve injury, thus avoiding complications^1^. In addition, IONM technology can promote neurophysiology research, reduce the learning curve for young surgeons, and help them grow rapidly^2^. The high incidence of thyroid diseases in China and the large proportion of neurological high-risk surgeries such as thyroid cancer have put forward higher requirements for laryngeal nerve protection^3^. IONM as an important auxiliary tool for nerve protection has gradually gained the attention of Chinese surgeons.

In recent years, IONM has been found widespread utility in thyroid surgery for the management of recurrent laryngeal nerve (RLN). IONM has gained popularity in China for both clinical and research applications. The Chinese Neural Monitoring Study Group (CNMSG) has also recommended the use of IONM for thyroid surgery^4^.

The CNMSG actively promotes IONM by organizing lectures at workshops, meetings, and conferences. The IONM technology is continuously undergoing diversification, with new techniques and approaches being introduced annually.

This survey-based study was designed to obtain information from endocrine surgery specialists on the general application and perioperative management for IONM in China. It aimed to identify the current practices of Chinese surgeons regarding the use of IONM in surgical patients.

## Materials and methods

### Ethics

The questionnaire-based study was approved by the Ethics Committee of China-Japan Union Hospital of Jilin University (Decision No. 2022-KYYS-078) and was conducted in accordance with the Declaration of Helsinki. Surgeons specializing in endocrine/thyroid surgery were asked to complete a questionnaire on a voluntary and anonymous basis.

### Inclusion and exclusion criteria

Included in the study were hospitals that had implemented IONM technology and had participated in online or offline neuromonitoring training activities organized by the CNMSG. Incomplete data were used as an exclusion criterion for this study. In the study of the percentage of malignant tumors we used as an exclusion criterion the surgery of tumors for which no postoperative pathology was performed or for which the pathology was benign. In the study of the incidence of postoperative hoarseness, we included preoperative vocal cord paralysis or failure to perform laryngoscopy as exclusion criteria.

### CNMSG

The CNMSG was founded in 2016 to investigate IONM tools, techniques, and technologies supporting Chinese thyroid surgeons. Over the years, CNMSG has emerged as a leading organization for Chinese surgery, as IONM is now used widely and is considered the gold standard for most thyroid surgeries. Therefore, the CNMSG has taken the responsibility for supporting innovation and introducing basic and advanced instruments, providing validation and guidance for their optimal and safe use.

### Questionnaires

At the beginning of CNMSG’s establishment, a survey on thyroid surgery in 37 university affiliated hospitals in China from 2011 to 2015 was conducted, including the number of thyroid surgeries, postoperative complications, the number of IONM applications and the proportion of malignant tumor surgery. Since 2015, the CNMSG has distributed national questionnaires among surgeons via e-mail and smartphone messaging applications.

In order to better grasp the development of neuromonitoring technology in China, the CNMSG members and experts discussed and developed a draft questionnaire, which was first used in a pilot study in some neuromonitoring training centers. The questionnaire was revised and improved according to the feedback and results of the pilot study, and the final version of the questionnaire for the official survey was finalized. These questionnaires include prospective questions, with one or more response options (Supplementary Files 1–4, Supplemental Digital Content 1, http://links.lww.com/JS9/D390, Supplemental Digital Content 2, http://links.lww.com/JS9/D391, Supplemental Digital Content 3, http://links.lww.com/JS9/D392, Supplemental Digital Content 4, http://links.lww.com/JS9/D393), related to five main topics: 1. Demographic/professional characteristics of participants; 2. Training methods for IONM; 3. Reasons for and frequency of conducting IONM; 4. IONM use and preferences in thyroid surgery; and 5. Scoring for interventional procedures/performance evaluation of IONM. This study compared the incidence of vocal cord paralysis before and after large-scale standardized use of IONM in China.

We invited the International Neuromonitoring Group to review the questionnaire and further tested the applicability of the questionnaire through a pilot study to validate the content validity of the questionnaire. In addition, we used Cronbach’s alpha coefficient to assess the degree of internal consistency of the questionnaire constructs. The Cronbach’s alpha of the questionnaire was 0.75, indicating that the questionnaire has good internal consistency and reliability.

### Data collection and processing

To ensure consistency among respondents, a rigorous standardized training was implemented in this study. First, all respondents had participated in an online or offline neuromonitoring training event organized by CNMSG, which provided detailed explanations of the questionnaire content and guidance on interpreting and filling out the questionnaire. In order to ensure the uniformity of data collection procedures, this study had developed a standardized operating procedure, in which members of the CNMSG organizing committee were involved in supervising and guiding every step of the questionnaire design, distribution, completion guidance, and data processing to ensure the reliability and authenticity of data collection. To minimize social desirability bias, the questions in the questionnaire were designed to be neutral and unbiased to reduce the impact on respondents’ answers. The questionnaires were designed with a clear time frame, and the questions were simplified and specified to reduce the burden of complex recall of past events on the respondents. In addition, a pilot study was conducted to verify whether the questions in the questionnaire were prone to recall bias, and the questionnaire was adjusted based on the results of the pilot study.

The survey participants in this paper are relevant professionals and hospitals engaged in research or practice in the field of neuromonitoring technology. Based on CNMSG, respondent selection was conducted by integrating multiple dimensions such as geographic distribution and professional background to ensure the diversity and comprehensiveness of the sample. A wider target group was convened by adopting a wide range of recruitment methods through multiple channels, such as professional associations, medical institutions, and academic conferences. Meanwhile, during the sample collection process, the representativeness of the sample is constantly evaluated to ensure that the sample represents the overall population or target group better by comparing the sample with the key characteristics of the target group (e.g. geographic distribution, professional background, etc.) to minimize the selection bias as much as possible.

### Statistics

Statistical analysis for this study data was performed using SPSS (Statistical Package for Social Sciences) 20 software (IBM Corp.). Descriptive statistical methods (means, standard deviations, and frequencies) were used to analyze the study results.

This work has been reported in line with the STROCSS criteria^5^.

## Results

### Significance of IONM use

**Figure 1:**
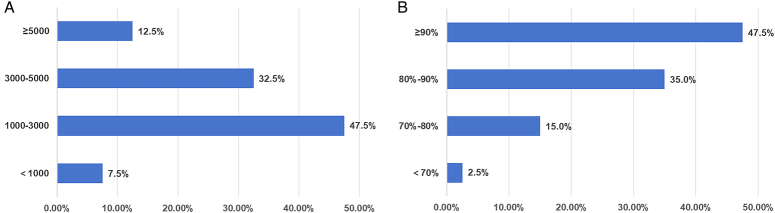
(A) The average annual surgical volume and (B) the proportion of malignant tumor surgeries performed in the surveyed hospitals.

The average annual thyroid surgery volume in the 40 tertiary hospitals exceeded 100 000 cases. Of these, 47.5% of the hospitals had an average annual thyroid surgery volume of 1000–3000 cases, 45% had an average annual volume of more than 3000 cases, and five (12.5%) hospitals performed more than 5000 thyroid surgeries (Fig. 1A). The average number of operations performed was 2710, well above the 2015 average of 1549.5 in 37 university hospitals. Malignant thyroid operations accounted for over 80% of the surgeries in 82.5% of the hospitals, and over 90% in 47.5% of the hospitals, with an overall percentage of malignant thyroid operations of 85.6% (Fig. 1B). The average percentage of malignant thyroid operations was 65.2% in 37 university hospitals in 2015 (Table 1), while in 2021, the average percentage was 84.2% in the 25 national IONM training centers.

**Table 1:** Thyroid surgery related data of 37 university affiliated hospitals in China in the past 5 years, in 2015.

Year	IONM cases	Surgical volume	Proportion of hoarseness (%)	Proportion of malignant (%)
2011	9.6	710.4	1.36	48.6
2012	27.1	863.0	1.27	55.2
2013	58.1	1041.9	0.96	60.4
2014	154.2	1360.8	0.98	63.8
2015	278.5	1549.5	1.04	65.2

IONM, intraoperative neuromonitoring.

**Figure 2:**
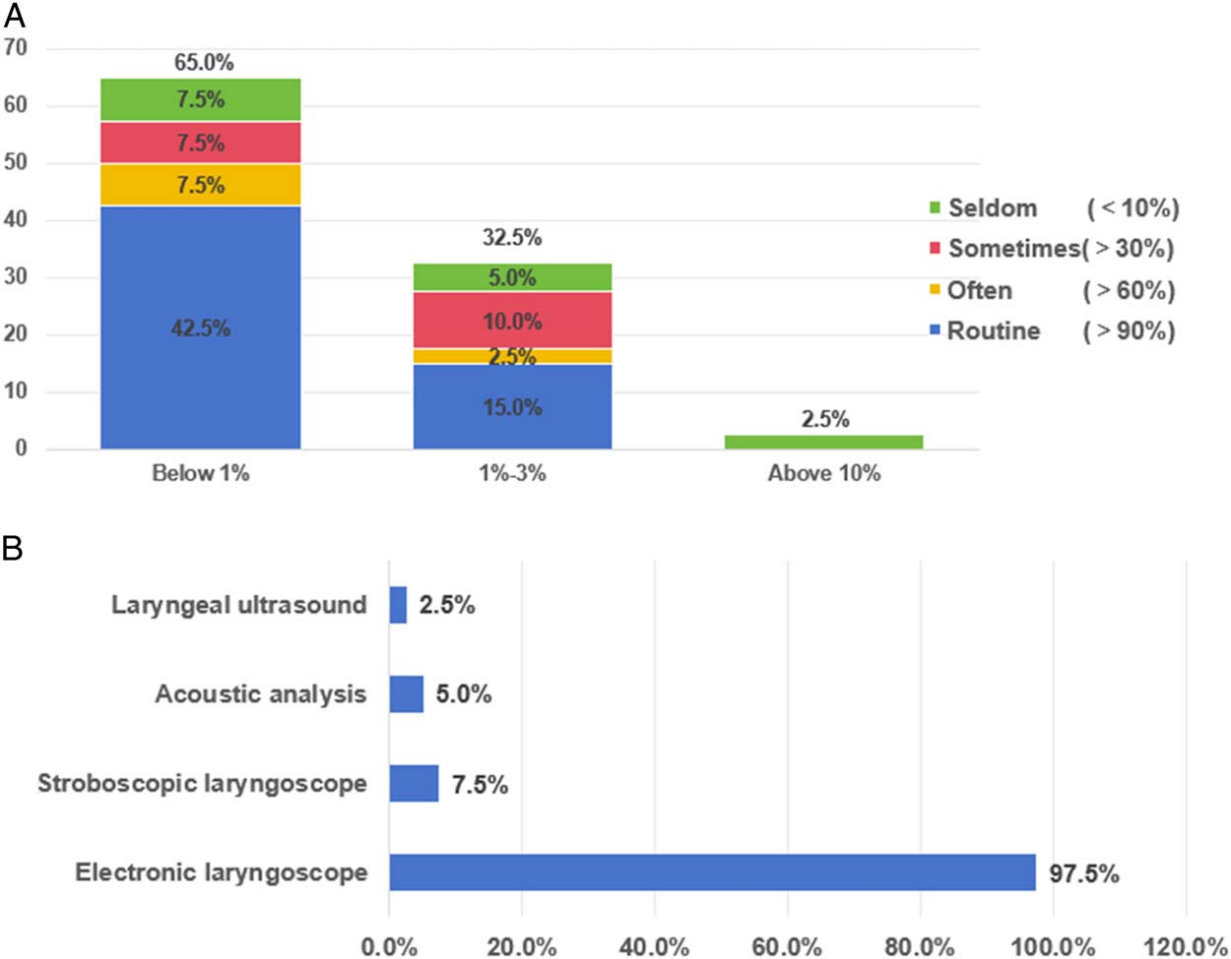
(A) Incidence of postoperative hoarseness and the application rate of IONM. (B) Methods for assessing vocal cord function in the surveyed hospitals.

The incidence of postoperative hoarseness reduced to less than 3% in more than 90% of the hospitals using IONM technology, and was less than 1% in 65% of the hospitals, with IONM use being more common in the hospitals with a lower incidence of postoperative hoarseness. Only one hospital (2.5%) used the IONM technique infrequently because of the cost (utilization rate<10%), resulting in a postoperative hoarseness incidence of greater than or equal to 10% (Fig. 2A).

Among the methods used for preoperative vocal fold assessment, electronic laryngoscopy was the predominant modality, used in 97.5% of the cases. Additionally, stroboscopic laryngoscopy, acoustic analysis, and laryngeal ultrasound were used in 3 (7.5%), 2 (5.0%), and 1 (2.5%) hospital, respectively (Fig. 2B). Preoperative laryngoscopy was routinely performed according to guidelines in 82.5% of the hospitals, while the protocol for postoperative laryngoscopy varied from hospital to hospital. Only 15% of the hospitals performed postoperative laryngoscopy routinely, 77.5% performed it as needed, while three (7.5%) opted not to perform it (Table 2).

**Table 2:** Perioperative vocal cord function assessment.

Assessment method	Proportion (%)
Routine preoperative and postoperative assessments	15.0
Routine preoperative assessments, with postoperative assessments as needed	60.0
Routine preoperative assessments without postoperative assessments	7.5
Preoperative and postoperative assessments as needed	17.5
No preoperative and postoperative assessments	0

### IONM application

**Figure 3:**
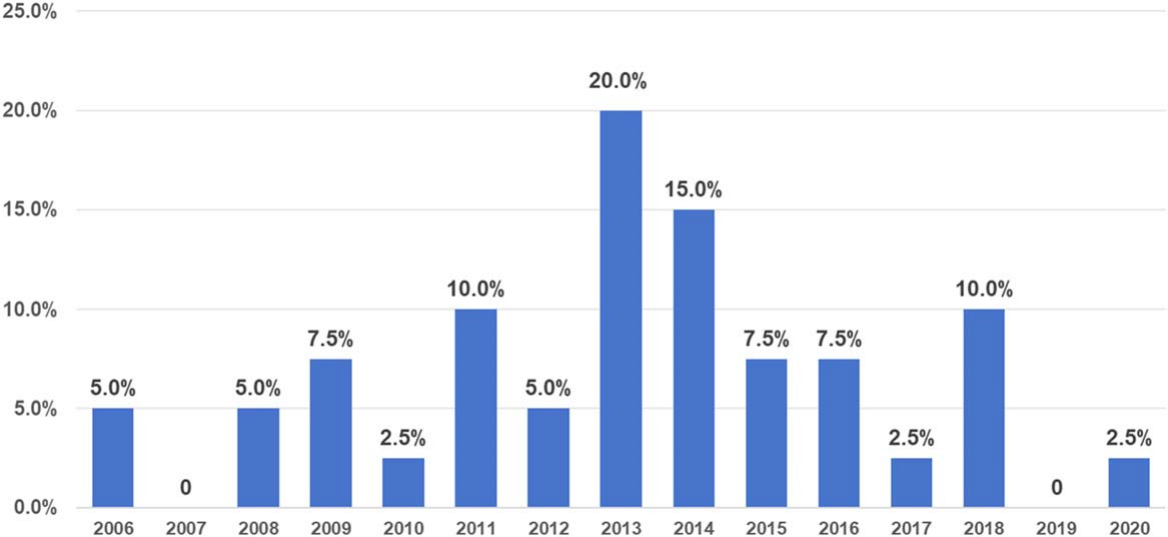
Year of intraoperative neuromonitoring introduction in the surveyed hospitals.

The surge in the application of IONM technology in China was initiated by the publication of the International Standards Guideline Statement in 2011^6^. This led to the adoption of IONM by hospitals for thyroid and parathyroid surgeries in accordance with international guidelines. IONM technology was adopted by 10% of the surveyed hospitals in 2011. Subsequently, the Chinese edition of neurophysiological monitoring guidelines for thyroid and parathyroid surgery was published in 2013, marking the start of nationwide clinical application of IONM. As a result, 65% of the surveyed hospitals adopted the IONM technology (Fig. 3).

**Figure 4:**
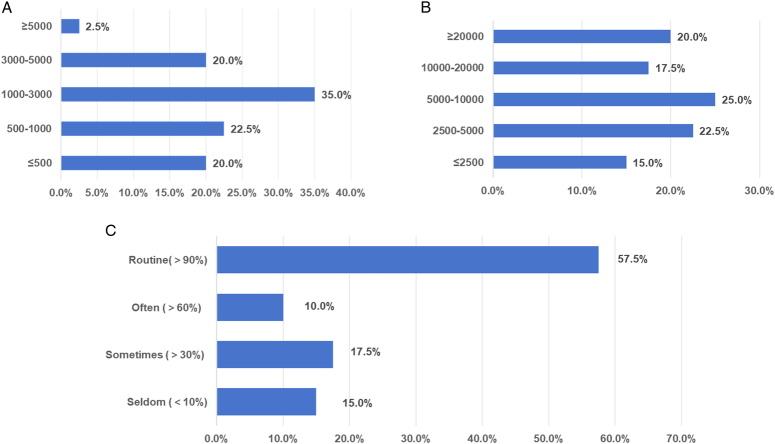
(A) Average annual number of cases, (B) cumulative number of cases, and. (C) proportion of the surveyed hospitals utilizing intraoperative neuromonitoring.

Among the surveyed hospitals, 80% applied IONM in more than 500 cases annually (18.5% the average volume), with nine (22.5%) hospitals applying it in more than 3000 cases (110.7% the average volume) on average annually (Fig. 4A). Moreover, 62.5% of the hospitals had a cumulative number of IONM applications exceeding 5000 cases (47.1% the average cumulative volume)., with eight (20%) hospitals accumulating over 20 000 cases (188.3% the average cumulative volume). (Fig. 4B). In terms of utilization rate, 57.5% of the hospitals achieved routine application (utilization rate > 90%; Fig. 4C).

**Figure 5:**
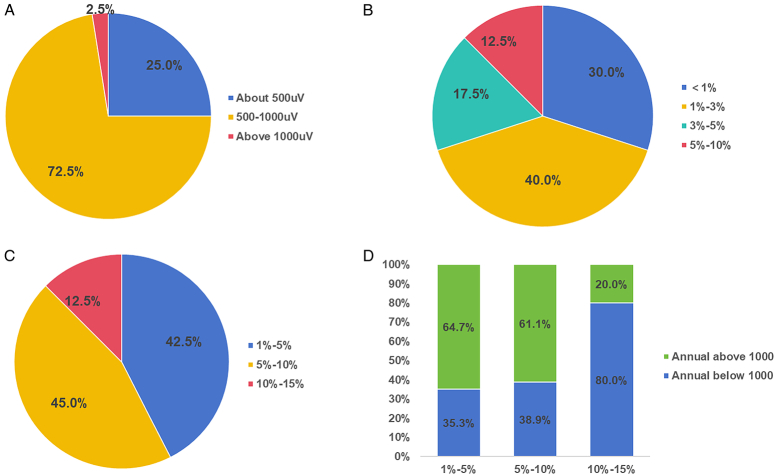
(A) Intraoperative V1 amplitude, (B) loss of signal ratio, (C) abnormal electromyography (EMG) ratio, and (D) proportion of annual intraoperative neuromonitoring cases with abnormal EMGs in the surveyed hospitals.

During surgery, 72.5% of the hospitals used V1 amplitudes of 500–1000 µV, while 25% reported V1 amplitudes around 500 µV (Fig. 5A). The intraoperative loss of signal (LOS) rate was below 3% in 70% of the hospitals, and 5–10% in five (12.5%) hospitals (Fig. 5B). In 87.5% of the hospitals analyzed, the intraoperative electromyography (EMG) signal abnormality rate was less than 10% (Fig. 5C). Notably, among the hospitals with a high EMG signal abnormality rate (10–15%), 80% used IONM in fewer than 1000 cases annually, whereas among those with a low abnormality rate (<10%), more than 60% used IONM in more than 1000 cases annually (Fig. 5D).

### Factors influencing IONM use

The main reasons for active adoption of the IONM technique by hospitals were its ability to identify dissociated nerves (92.5%) and reduce nerve injury (95%) (Table 3). Meanwhile, the main limitation for its use was the cost (72.5%), followed by the lack of cooperation from anesthetists (15%) (Table 4).

**Table 3:** Rationale for IONM application.

Reasons	Proportion (%)
Identifying dissociated nerves	92.5
Reducing the risk of nerve damage	95.0
Shortening the duration of surgery	57.5
Shortening the learning curve of young doctors	65.0
Medical litigation needs	20.0
Others	5.0

IONM, intraoperative neuromonitoring.

**Table 4:** Rationale for refraining from IONM.

Reasons	Proportion (%)
The technology has not been introduced yet	0
Lack of anesthesiology cooperation	15.0
Clinician inexperience	0
Cost	72.5
Patient preference	10.0
Lack of limits	15.0
Others	15.0

IONM, intraoperative neuromonitoring.

### IONM training

In 2016, the establishment of the CNMSG in Shanghai led to an increase in the number of national IONM training centers from 12 to 25, with membership increasing to 97 individuals. The CNMSG has published several academic monographs and training materials, organized numerous national training courses, and delivered over 200 lectures at the grassroots level. In China, more than 20 000 individuals have been trained in IONM. In the 2016 IONM survey, the average correction rate for all questions was 68.2% ± 22.9%, with the average correction rates for basic and advanced knowledge being 74.4% and 57.8%, respectively.

**Figure 6:**
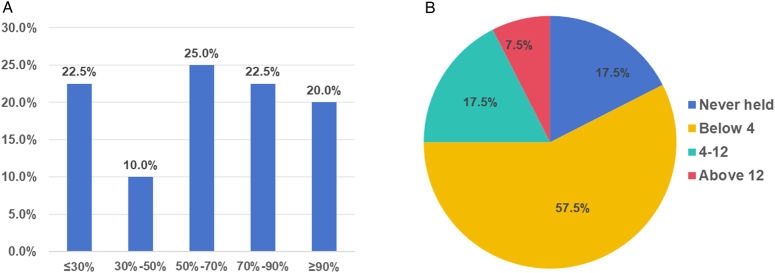
(A) Proportion of intraoperative neuromonitoring (IONM)-trained personnel and (B) the number of IONM. training activities carried out in the surveyed hospitals.

Specialized IONM training was provided to more than 50% of thyroid surgical team members in 67.5% of the surveyed hospitals, while in 7 (17.5%) hospitals, all thyroid surgical team members were trained in IONM. However, 22.5% of the hospitals had less than 30% of the team members trained in this area (Fig. 6A). Additionally, 57.5% of the hospitals provided fewer than four training sessions on IONM, 3 (7.5%) hospitals offered more than 12 sessions, and 7 (17.5%) hospitals had never provided such training (Fig. 6B). Primary areas that require further strengthening included the methods and directions of IONM scientific research and the analysis and processing of causes of EMG signal abnormalities, each accounting for 72.5% of the total. Moreover, we identified a need to deepen the understanding of the basic IONM knowledge (Table 5).

**Table 5:** The content of training to be strengthened.

Content	Proportion (%)
IONM principle, parameters, and equipment	50.0
IONM system establishment and setup	35.0
Key points of IONM operation	45.0
Analysis and treatment of abnormal electromyography signals	72.5
Methods and directions for carrying out scientific research	72.5

IONM, intraoperative neuromonitoring.

### Scientific research on IONM

**Figure 7:**
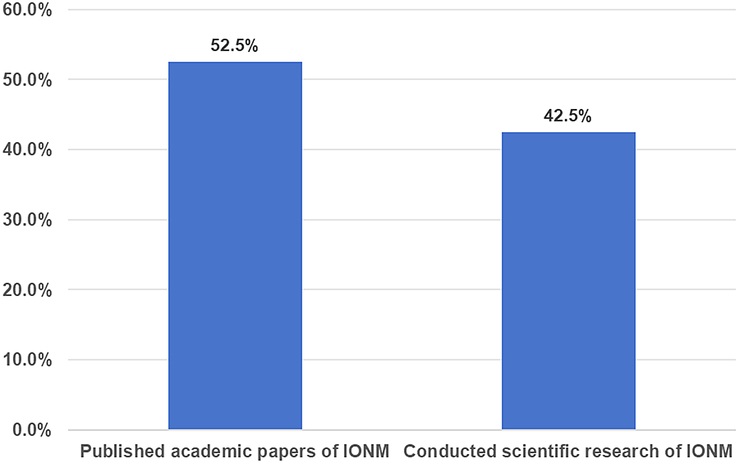
Proportion of research conducted and papers published in the surveyed hospitals.

Among the surveyed hospitals, 42.5% had conducted research on IONM technology and 52.5% had published studies related to IONM (Fig. 7). The 433 surgeons from 25 IONM training centers recommended that future IONM research should focus on technological advancements.

## Discussion

Postoperative complications of thyroid surgery include vocal cord paralysis due to injury of the recurrent laryngeal nerve^7^, postoperative hypocalcemia due to impaired parathyroid function^8^, postoperative hemorrhage and lymphatic leakage. For patients with lateral cervical lymph node metastases, lateral cervical lymph node dissection may result in cervical facial swelling and peripheral cervical numbness and pain^9^. The complications of thyroid surgery affect the quality of life of patients, so the occurrence of complications should be reduced by detailed preoperative examination and improvement of intraoperative operation.

Over the 15 years since the introduction of IONM in China, the landscape of thyroid and parathyroid surgery has undergone significant transformation. Initially focused on avoiding nerves, surgical approaches have evolved to include precise dissection and identification of nerves. Currently, IONM can be used to localize, identify, visualize, and protect the nerves, making it an indispensable component of modern thyroid and parathyroid surgery.

The findings of this survey revealed that the average annual volume of thyroid surgery across 40 tertiary hospitals exceeds 100 000 cases. This increase in surgical volume can be attributed to three factors. Firstly, China’s large population base combined with an increasing incidence of thyroid diseases contributed to a growing demand for surgical intervention. Secondly, advancements in diagnostic accuracy have led to higher detection rates for thyroid diseases requiring surgical management. Thirdly, the development and popularization of novel technologies and concepts, including IONM, have accelerated the learning curve for young doctors, facilitating rapid professional growth and increasing the surgical volumes in hospitals. As a result of the increase in surgical volumes and patient expectations for improved quality of life, IONM has emerged as an important tool for neuroprotection in thyroid surgery.

Globally, the annual incidence rate of thyroid cancer has been reported as ~5.4% in males and 6.5% in females^10^. The National Cancer Centre of China has reported a standardized incidence rate for thyroid cancer in China of 14.65 per 100 000 person-years, ranking seventh among the malignant tumors in the country^3^. Over the recent years, the incidence of malignant thyroid tumors has remained high. Advancements in preoperative precision diagnosis and treatment have led to improved detection rates and timely surgical interventions for a greater number of malignant tumor patients. Standardized thyroid surgery practices across various institutions and the utilization of non-surgical treatments, such as radiofrequency ablation, for non-malignant tumors have also contributed to the increase in the proportion of operations for malignant tumors.

Vocal cord paralysis is a serious complication of thyroid surgery, commonly resulting from intraoperative RLN injury. The criteria for recurrent laryngeal nerve injury are the presence of hoarseness and postoperative laryngoscopy findings of altered vocal cord motion. According to the recovery time of hoarseness, the recurrent laryngeal nerve injury can be categorized into temporary injury (recovery within 6 months) and permanent injury (no recovery at 6 months). According to the location of the recurrent laryngeal nerve injury, it can be categorized into type I injury (segmental injury, and the point of nerve injury can be found by IONM) and type II injury (total injury, and there is no clear point of injury)^8^. The incidence rates for transient and permanent RLN injury are 2–11% and 0.6–1.6%, respectively, influenced by surgeon experience, anatomical variations, and the thyroid condition^11,12^. Several studies have demonstrated the positive role of IONM in reducing RLN injuries^13,14^, particularly in complex procedures.

Statistical analysis from 2011 to 2015 (Table 1) found that the application rate of IONM was low in China before 2012, and the proportion of postoperative hoarseness, one of the serious complications of thyroid surgery, was more than 1%. However, with the increasing number of surgeries, the proportion of difficult surgeries such as malignant tumor surgery is increasing, and the proportion of postoperative hoarseness is decreasing. After the introduction of IONM technology in 2013, the proportion of postoperative hoarseness decreased by 30% compared with that in 2011. The survey results in 2023 showed that 65% of the surveyed hospitals had less than 1% postoperative hoarseness, and with the increase of the incidence of hoarseness, the proportion of hospitals with low IONM utilization rate was increasing. (Fig. 2A).

Preoperative laryngoscopy allows motor assessment of a patient’s vocal cords, reflecting the neurological function. Laryngoscopy (L1, L2) is important for an objective assessment of RLN injuries, optimization of surgical approaches, reducing risks, and delineating individual responsibilities^4,15^. The 2016 survey revealed that nearly 80% of the surveyed hospitals opted for routine preoperative laryngoscopy, 45% performed postoperative laryngoscopy as necessary, while 10% did not routinely perform pre- or postoperative laryngoscopy. Some hospitals cited concerns about the potential for postoperative laryngoscopy to highlight asymptomatic or undetectable vocal cord movement abnormalities, potentially increasing the risk of medical disputes, explaining the reduced frequency of use in 2016. Nowadays, hospitals acknowledge the importance of laryngoscopy, with preoperative assessments performed routinely. However, owing to the high cost, postoperative laryngoscopy is performed only when a reassessment of vocal cord motor function is necessary. To address the patients’ vocal needs, some hospitals have begun using stroboscopic laryngoscopy and acoustic analysis to improve the diagnostic sensitivity and provide a reliable basis for comparing pre and postoperative vocal cord functions^16,17^.

Data show that IONM adoption and development varies in different regions. IONM technology is highly utilized in Germany (90%), the United States (80%), and Australia (80%)^18,19^, but less so in the United Kingdom (24%), Italy (14%) and Poland (8%)^20–23^. The results of a global surgical survey from six continents showed that of the 1015 respondents, 83% had used IONM (65.1% for regular use and 18.1% for selective use). Among selective users, most reported using IONM in reoperation cases (95.1%) and in cases of preoperative vocal cord paralysis (59.8%)^24^.

The survey data showed the widespread acceptance and high utilization rate of IONM technology among hospitals. Over 60% of the respondents utilized IONM only for complex operations, while routine usage remained below 30% in 2016. However, by 2021, survey results from 25 IONM training centers indicated that IONM usage rates exceeded 80% in 72% of these centers. Among these training centers, where staff are more professionally trained and operate in a standardized manner, earlier adoption and higher utilization rates of IONM were observed.

The key to realizing the full benefits of IONM lies in the implementation of standardized operating procedures. Preoperatively, the amplitude of the vagal signal V1 serves not only to confirm successful establishment of the IONM system^4^, but also facilitates early identification of non-returning recurrent nerves, reducing the risk of nerve injury. International IONM guidelines recommend that the initial V1 amplitude should be greater than 500 µV^6^. Data from a recent IONM study of 460 patients with a high risk of RLN thyroid surgery showed that the incidence of postoperative vocal cord paralysis was significantly reduced when the initial amplitude was higher^25^. In this survey, 100% of the hospitals reported intraoperative V1 amplitudes exceeding this threshold, indicating successful setup of monitoring systems in accordance with the guidelines.

An intraoperative EMG abnormality is defined as a sudden and significant change in the EMG during the procedure after obtaining a satisfactory signal, while LOS is defined as fall in amplitude below 100 µV. If intraoperative LOS occurs in a single thyroid gland during bilateral thyroid surgery, neurological function should be carefully assessed and staged surgery should be performed if necessary^26^. Hospitals with lower average annual IONM cases are more susceptible to intraoperative EMG abnormalities due to suboptimal application of the IONM technology. Studies have reported that the incidence of EMG signal abnormalities resulting from non-standardized device use or surgical procedures ranges from 3.8 to 23%^6,27,28^. Pseudo-EMG signal loss during surgery, which is unrelated to nerve injury, can psychologically influence the surgeon, impede the surgical process, and even lead to erroneous surgical decisions. Therefore, strict adherence to the updated clinical guidelines is required during surgical procedures to reduce the incidence of EMG signal abnormalities resulting from system failures.

The primary reasons for hospitals adopting the use of IONM were early nerve warning and protection, neurological function assessment, and high sensitivity for rare neurodegenerative conditions. Survey results from 2016 showed that the main advantages of IONM technology were real-time nerve localization and intraoperative assessment of nerve function, followed by shortened operation times and predicting postoperative vocal cord paralysis. These findings were in agreement with the current survey results. At present, IONM technology is not associated with hardware issues and there are several clinicians proficient in its application. However, its cost remains the primary obstacle hindering further development of this technology globally and in China. In the United States, the cost of applying IONM surgery increased by $387^29^. In Korea, the cost of applying the IONM technique was significantly higher than that of normal surgery ($328.2 ± $220.1 vs. $278.7 ± $141.4, *P*<0.05)^30^. In Italy, the hospitalization cost of the IONM technique was 5–7% higher than that of conventional thyroidectomy^31^. And the cost in China was about $600 in addition. The gradual introduction of the DRG payment model in China has forced some hospitals to abandon the use of IONM. Additionally, this survey demonstrated that the current IONM training activities focus primarily on surgeons while neglecting anesthetists. Anesthesia plays a crucial role in setting up the IONM system and acquiring effective neural EMG signals, particularly in fixing the catheter electrode and administering neuromuscular blockers, which directly affect intraoperative EMG signal quality^32^. Therefore, future training and teaching efforts should focus on the collaboration between anesthetists and surgeons.

In 2021, the International Neural Monitoring Study Group (INMSG) developed an international IONM training curriculum to provide surgeons with professional guidance^33^. Systematic and standardized training not only accelerates the learning curve for young surgeons but also enhances surgical procedures and reduces the incidence of RLN injuries using IONM^34^. Studies have indicated that proficiency in the IONM technique requires an experience of at least 50–100 cases involving IONM application^35^

This questionnaire survey revealed that more extensive training measures were implemented in IONM training centers, while fewer measures were implemented in non-training centers due to the lack of experience and trainers. The learning content requiring emphasis in 2023 differed from that in the 2018 survey, which demonstrated an urgent need for basic operation, troubleshooting, and other basic theoretical and practical topics in neuromonitoring. However, the current survey demonstrated a reduced need for focusing on these topics. With the nationwide IONM training initiatives, hospitals have gradually acquired basic theoretical knowledge of IONM, and the focus of future training has shifted toward scientific innovation and analysis of special intraoperative situations in IONM. To address these issues, IONM training institutions should serve as comprehensive academic exchange centers, facilitating the dissemination of the latest research advancements and promoting IONM technology development in surrounding regions. Concurrently, learning activities should also focus on the latest edition of the guidelines. The 2023 Guidelines for Intraoperative Neuromonitoring in Thyroid Surgery systematically analyze and classify the causes of abnormal intraoperative EMG signals, propose solutions, and provide detailed explanations of IONM principles, parameters, and standard operating procedures. Enhancing knowledge of the latest guidelines can significantly improve the effectiveness of neuromonitoring applications^4^.

The establishment of CNMSG in 2016 led to the creation of a learning and sharing platform for IONM in China. This initiative has yielded numerous scientific breakthroughs in hardware design^36,37^, software development^38^, and endoscopic robotic surgical applications^39^. The focus of research has shifted from retrospective to prospective studies, with the CNMSG issuing expert consensus reports on the external branches of superior thyroid laryngeal nerve in 2017 and robotic thyroid surgery in 2019. Medical institutions and research clinics are gradually transitioning from purely clinical applications to scientific research endeavors. Despite advancements in monitoring equipment and mature monitoring technology, challenges still persist, necessitating further scientific research.

This paper is the first investigation of the current state of IONM application in China, but there are some limitations. Firstly, as with all survey studies, it is subject to respondent bias. Although we have taken a series of measures to reduce the bias, sample bias and measurement bias still exist. Secondly, this survey did not investigate other complications of thyroid surgery in detail and lacked data on thyroid surgery before IONM application. In addition, the sample size needs to be further expanded due to the differences in the level of medical care between regions in China. Although we tried our best to expand the sample size, we still may not be able to cover hospitals and physicians in all regions of the country. Also there may be differences in the implementation of IONM technology in different regions and hospitals, and these differences may affect the results of our study.

From both clinical application and scientific research perspectives, the future trajectory of IONM technology is expected to evolve in the following ways:

1. Realization of Continuous Intraoperative Neuromonitoring (C-IONM): Currently, intermittent intraoperative neuromonitoring is prevalent in China. However, it only intermittently evaluates nerve function integrity, leaving the nerve vulnerable to injury between assessments. In contrast, C-IONM offers continuous intraoperative monitoring of nerve function, providing early warning of injury risks and allowing surgeons to promptly adjust procedures for preventing permanent vocal cord paralysis^40^.
2. Improvements in EMG signal stability: The quality of EMG signals is fundamental for effective IONM. The influences of anesthesia regimen, catheter positioning, and patient variability can cause intraoperative EMG signal fluctuations, affecting IONM efficacy. To improve signal stability, research is needed on optimizing anesthesia protocols and developing more stable receiving electrodes.
3. Establishing sensitive and reliable parameter indicators: Currently, the most important parameter indicators include amplitude, latency, and duration. However, exploring new indicators, such as area under the waveform, holds promise in the era of artificial intelligence, potentially enabling accurate and intelligent nerve injury predictions.
4. Technology advancements for endoscopic and robotic thyroid surgery or ablation: Endoscopic and robotic DaVinci thyroid surgeries are gaining popularity due to their unique advantages. However, few studies have investigated the application of endoscopic thyroid IONM in these procedures. Areas requiring further exploration include RLN injury mechanisms, C-IONM applications, troubleshooting methods, standardization of surgical protocols, and stimulation electrode selection. Integration of the endoscopic and IONM technologies offers a promising direction for future developments.

## Conclusions

Based on findings from the Chinese national neuromonitoring questionnaire survey, this study analyzed the practical application and challenges of IONM technology in thyroid surgeries in China across three key aspects, including clinical application, education, and scientific research, and proposed possible directions for future development. Currently, IONM technology is experiencing rapid growth, but regional disparities persist, and scientific research efforts remain insufficient. These areas will be pivotal for future development endeavors. In future, high-quality advancements in IONM technology require diversified training formats, enhanced training content, expanded training scope, and elevated training standards.

## Ethical approval

The study design was approved by the Ethics Committee of Jilin University (Decision No. 2022-KYYS-078).

## Consent

Written informed consent was obtained from the patient for publication of this case report and accompanying images. A copy of the written consent is available for review by the Editor-in-Chief of this journal on request.

## Source of funding

This study was initiated by the Chinese Neural Monitoring Study Group (CNMSG). This study was sponsored by the Science and Technology Research Project of Education Department of Jilin Province, China, [No. JJKH20221065KJ]; Jilin Province Health Research Talent SpecProject [No. 2020SCZ03]; Beijing Cihua Medical Development Foundation [J2023107004].

## Author contribution

Conceptualization: H.S. and Y.S.Z.; methodology: J.D.K.,CL.L., F.L., and R.D.; formal analysis: P.Y.W. and YS.Z.; investigation, J.D.K. and P.Y.W.; resources: J.D.K., Y.S.Z., P.Y.W., and F.L.; writing—original draft preparation: P.Y.W. and YS.Z.; writing—review and editing: H.S., G.D. supervision: H.S.; project administration: Y.S.Z., and H.S.; funding acquisition: Y.S.Z. and H.S. All authors have read and agreed to the published version of the manuscript.

## Conflicts of interest disclosure

The authors declare no conflicts of interest.

## Research registration unique identifying number (UIN)

The unique identifying number is: researchregistry10137

## Guarantor

All authors have read and agreed to the published version of the manuscript.

## Data availability statement

Please refer to the corresponding author when necessary.

## Provenance and peer review

Not applicable.

## Supplementary Material
