# Supplementary material for: Utilization of recurrent laryngeal nerve monitoring during thyroid surgery in China: a point prevalence survey (2015–2023)
Source: Int J Surg. 2024 Sep 6;111(1):439–49. doi: 10.1097/JS9.0000000000002084 (PMC11745604; doi:10.1097/JS9.0000000000002084)
Supplement: Supplementary file 1 [file js9-111-0439-s001.doc]

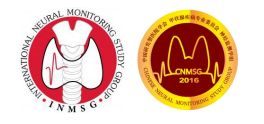
**Questionnaire for the First Advanced Training Course on Neuromonitoring in China**

In order to better understand the participants' understanding of intraoperative neural monitoring (IONM) technology, please fill out the following questionnaire for the first China Advanced Training Course on Neuromonitoring, and thank you again for your support and feedback.

Name: Affiliations: Department: E-mail:

**1. Which of the following is correct regarding laryngoscopy indications?**

□Preoperative and postoperative laryngoscopy are performed in all cases

□Postoperative laryngoscopy is performed in all cases

□Preoperative laryngoscopy is performed in all cases

□Preoperative and postoperative laryngoscopy would be performed only in high-risk cases

**2. As recommended by the Neural Monitoring Study Group, IONM can be applied to:**

□Assist dissection to expose nerves

□Identify the recurrent laryngeal nerve (nerve mapping)

□Predict postoperative neurological function and identify the injury point

□All of the above

**3.** **Which of the following is correct about vagus nerve stimulation?**

□It should be done after dissection of the free thyroid gland

□The accuracy of evaluating glottic function is worse than that of direct laryngeal nerve stimulation

□The integrity of the entire neural circuit can be tested

□Stimulation of the distal of the lesion can produce false negative results

**4. The most reliable methods of recurrent laryngeal nerve monitoring include:**

□Audio and image system based on monitoring catheter recording electrodes

□Image system and glottic observation based on needle recording electrodes

□Audio system and laryngeal palpation based on needle recording electrodes

□Image system and glottic pressure monitoring system based on monitoring catheter recording electrodes

**5. Which of the following is correct about the advantages and disadvantages of bipolar stimulation probe evaluation?**

□It cannot be integrated into anatomical instruments

□It generates more diffuse current than monopolar electrodes

□It is less sensitive than monopolar electrodes

□It is not suitable for locating nerve course

**6. Which additional anesthetics should the anesthesiologist avoid during neuromonitoring?**

□Muscle relaxants (succinylcholine, rocuronium bromide, atracurium)

□Inhalational anesthetics (isoflurane, desflurane)

□Opioid anesthetics (remifentanil, fentanyl, sufentanil)

□Propofol

**7. Which muscle relaxant is more appropriate for tracheal intubation in patients with pseudocholinesterase deficiency?**

□Long-acting nondepolarizing muscle relaxants

□Depolarizing muscle relaxants

□Short-acting nondepolarizing muscle relaxants

□Pseudocholinesterase deficiency does not affect the duration of neuromuscular blockade

**8. Which of the following is correct about endotracheal tube recording electrodes?**

□It should be placed at the subglottic level

□It belongs to the stimulation side of the monitoring system

□It belongs to the recording side of the monitoring system

□It is embedded on the surface of the tracheal tube

**9. Which of the following is correct about monitoring catheter intubation?**

□The correct position of the monitoring catheter is necessary to ensure good contact between the vocal cords and the recording electrodes

□The monitoring catheter should be slightly smaller than the size of a regular tracheal tube

□Cores should not be used when placing neuromonitoring catheters

□Only lidocaine gel could be lubricated during the intubation of the monitoring catheter

**10. Which** **devices should be kept away from neuromonitoring devices?**

□Ultrasonic devices (Harmonic Focus)

□Vacuum aspirator

□Radio frequency devices（Ligasure）

□Monopolar electrotome

**11. Which of the following is correct about monitoring catheter immobilization?**

□It is immobilized after the patient's neck has been hyperextended and the position has been placed

□The patient's neck is not required to be in the hyperextension position when immobilizing the monitoring catheter

□The monitoring catheter should not be immobilized to facilitate adjustment at any time during the procedure

□It is immobilized after the patient's neck is in the hyperextension position and before the position is placed

**12. Which of the following is correct about impedance values?**

□It has nothing to do with recording EMG signals

□It should be detected after dissecting the free thyroid gland

□It can indicate the correct position of the monitoring catheter

□It can only indicate to record electrode contact with the patient

**13. When examining monitoring parameters, electrode impedance should:**

□Single electrode impedance should be greater than 5 kΩ.

□Single electrode impedance value should be less than 5 kΩ, while the impedance difference value is less than 1kΩ.

□Single electrode impedance value should be less than 5 kΩ, while the impedance difference value is less than 5kΩ.

□Single electrode impedance value should be less than 1 kΩ, while the impedance difference value is less than 1kΩ.

**14. What does it indicate when the impedance difference is high？**

□Poor contact between electrode and patient requiring readjustment of the monitoring catheters

□The monitoring catheter model is appropriate

□The electrodes are in good contact with the patient and the procedure is allowed to begin

□The monitoring catheter model is improper

**15. The first detection in the operative field is:**

□Translaryngeal detection and/or stimulation of the strap muscles

□Translaryngeal detection only

□Unable to perform preliminary stimulation test before detecting V1 signals

□Stimulation of the strap muscles only

**16. Why do we need to detect the V1 signal?**

□To verify that the nervous system is well established

□V1 can be used as an important reference value for subsequent anatomical operations

□To verify that the monitoring catheter is positioned correctly

□All of the above

**17. Why do we need to detect the V2 signal?**

□To verify the integrity of the recurrent laryngeal nerve

□V2 can be used as a reference for troubleshooting the monitoring system

□To distinguish between type 1 and type 2 injuries

□All of the above

**18. Which of the following is an IONM standardized operational procedure?**

□L1–V1–R1–R2–V2–L2

□V1–R1–R2–V2

□L1 – R1 – V1 – V2 – R2 – L2

□L1–L2–V1–V2–R1–R2

**19. When the RLN is detected without an EMG signal amplitude, or with an** **amplitude below 100 µV, we should :**

□Stimulate the ipsilateral vagus nerve and observe for the presence of a laryngeal muscle twitch response, the malfunction occurs on the recording side if there is no response

□Stimulate the ipsilateral vagus nerve and observe for the presence of a laryngeal muscle twitch response, the malfunction occurs on the recording side if there is response

□Stimulate the ipsilateral vagus nerve and observe for the presence of a laryngeal muscle twitch response, the malfunction occurs on the stimulation side if there is response

□Stimulate the contralateral recurrent laryngeal nerve and observe for the presence of a laryngeal muscle twitch response, the malfunction occurs on the recording side if there is response

**20. If stimulation of one side of the recurrent laryngeal nerve does not produce a large enough EMG signal, but the** **laryngeal muscle twitch response is present, then the contralateral vagus nerve is stimulated:**

□Producing a low EMG signal indicates a possible recording side malfunction

□Producing a high EMG signal indicates a possible recording side malfunction

□Stimulation with >10mA may be effective

□It is not important to detect the EMG signal of the contralateral vagus nerve during bilateral total thyroidectomy

**21. What does it indicate if the laryngeal muscle twitch disappears?**

□The malfunction occurs on the recording side

□The malfunction occurs on the stimulation side

□Non-recurrent laryngeal nerve can be excluded

□Recurrent laryngeal nerve injury is verified

**22. When EMG signal is undetectable or lower than**  **, suggesting the possibility of loss of signal (LOS)**

□＜10 µV

□＜500 µV

□＜1000 µV

□＜100 µV

**23. Loss of signal (LOS) is defined as:**

□No laryngeal muscle twitch response or glottic movement could be detected

□The EMG signal is lower than the initial signal

□No signal or poor signal (e.g.100µV or less) after stimulating by 1mA current stimulation with a clean surgical field

□All of the above

**24. The correct recurrent laryngeal nerve stimulation current is:**

□1A

□0.1 mA

□1 mA

□10 mA

**25. The criteria for determining loss of signal (LOS) is:**

□Laryngeal muscle twitch becomes smaller

□It can be evaluated only if the initial EMG signal is good

□The initial EMG signal is not obtained

□The EMG signal waveform is lost

**26. The International Neural Monitoring Study Group recommends that the initial value of V1 signal should be:**

□＞500 µV

□＜500 µV

□＜100 µV

□100-300 µV

**27. When the nerve signal is lost, if the contralateral vagus nerve EMG signal is not detected:**

□It is necessary to perform staged surgery

□It may be caused by the poor position of the monitoring catheter

□It may be caused by bilateral nerve injury

□Loss of neural signals may indeed have occurred

**28. When one side of the nerve signal is lost, the nerve injury point should be located:**

□Type 2 injury refers to the total injury of bilateral recurrent laryngeal nerve and vagus nerve

□Type 2 injury is mostly caused by thermal injury

□Type 1 injury refers to the entire injury, and type 2 injury refers to the presence of damage points

□Type 2 injury refers to the entire injury, and type 1 injury refers to the presence of damage points

**29.Which of the following belongs to the malfunction of the stimulation side?**

□Transtracheal stimulation (stimulus artifact)

□Fault of probe

□Insufficient stimulating current

□All of the above

**30. Which of the following is correct about the amplitude of the nerve EMG signal?**

□It varies greatly from patient to patient

□It changes only in response to nerve injury

□It remains stable during neuromonitoring

□It rises only when the stimulus current intensity (mA) increases

**31. The stimulus threshold is defined as:**

□The intensity of the stimulating current that can induce the EMG signal

□The intensity of the lowest stimulating current that can induce the earliest EMG signal

□The intensity of the stimulating current that can induce the physiological EMG signal at the earliest time

□The intensity of the highest stimulating current that can induce the earliest EMG signal

**32. Which of the following is correct about the latency?**

□It represents the number of nerve fibers involved in the depolarizing response

□It depends on the distance between the stimulation point and the contralateral vocal fold

□It depends on the distance between the stimulation point and the ipsilateral vocal fold

□It represents the speed or ease with which the nerve fibers are depolarized

**33. False positive results for nerve injury are defined as:**

□The nerve signal is loss, with bilateral vocal cord paralysis

□The nerve signal is loss, vocal cord movement is good

□The nerve signal is good, but bilateral vocal cord paralysis

□The nerve signal is loss, with unilateral vocal cord paralysis

**34.** **Which of the following is not the cause of a false positive result?**

□The nerve is covered by blood or fascia

□Monitoring catheter displacement

□Early neurological recovery after vocal cord paralysis

□EMG signals are obtained by detecting the distal to the nerve injury point

**35. Which of the following is not the cause of a false negative result?**

□Damage that occurs after the last probe

□Loss of signal due to muscle relaxants

□The posterior branch of the nerve is injured

□Late-onset neurapraxia
