# Supplementary material for: Utilization of recurrent laryngeal nerve monitoring during thyroid surgery in China: a point prevalence survey (2015–2023)
Source: Int J Surg. 2024 Sep 6;111(1):439–49. doi: 10.1097/JS9.0000000000002084 (PMC11745604; doi:10.1097/JS9.0000000000002084)

**
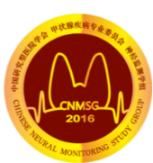

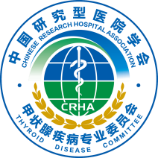
Chinese Neural Monitoring Study Group（CNMSG）Third Questionnaire**

In order to better understand the current status of the application of intraoperative neural monitoring (IONM) for thyroid surgery in China, promote the standard operation and popularize the practical skills, please fill in the "Chinese Neural Monitoring Study Group（CNMSG）Third Questionnaire" carefully. Thank you again for your active participation and look forward to your valuable suggestions to assist the group to make future work plans!

Name: Gender: □man □woman Age: E-mail:

Affiliations: Department: Tel:

Professional title:□Chief physician □Associate chief physician

□Attending physician □Physician

Please complete the following table according to the data of thyroid surgery from

January 2016 to December 2016:

| **Thyroid surgery** | **Open surgery** | **Endoscopic surgery** | **Robotic surgery** | **Total surgery** |
| --- | --- | --- | --- | --- |
| **Thyroid surgery volume** |  |  |  |  |
| **Proportion of thyroid cancers (%)** |  |  |  |  |
| **Proportion of I-IONM (%)** |  |  |  |  |
| **Proportion of C-IONM (%)** |  |  |  |  |
| **IONM indicators: (multiple choices) (%)** | □L1□V1  □R1□R2  □V2□L2 | □L1□V1  □R1□R2  □V2□L2 | □L1□V1  □R1□R2  □V2□L2 | **/** |
| **Proportion of system malfunction (%)** |  |  |  |  |
| **Proportion of recurrent laryngeal nerve injury (%)** |  |  |  |  |
| **Proportion of temporary injury**  **(recovered within 6 months) (%)** |  |  |  |  |
| **Proportion of permanent injury (%)** |  |  |  |  |
| **Proportion of postoperative hypocalcemia (%)** |  |  |  |  |
| **Proportion of temporary hypocalcemia (recovered within 6 months) (%)** |  |  |  |  |
| **Proportion of permanent** **hypocalcemia injury (%)** |  |  |  |  |

| **Central lymph node dissection in thyroid cancer** | **Open surgery** | **Endoscopic surgery** | **Robotic surgery** |
| --- | --- | --- | --- |
| **Central lymph node dissection volume** |  |  |  |
| **Proportion of I-IONM (%)** |  |  |  |
| **Proportion of C-IONM (%)** |  |  |  |
| **Whether V1 and V2 are retested before and after central lymph node dissection** | □Yes  □No | □Yes  □No | □Yes  □No |

IONM: Intraoperative neural monitoring
C-IONM: Continuous intraoperative neural monitoring
I-IONM: Intermittent intraoperative neural monitoring

**1. Your learning curve of I-IONM:**

Learning methods (multiple choices):

□The study class □Conference □Self-study

Volume: □10 □50 □100 □＞100 Time (year): □1 □2 □＜5 □＞5

**2. Your learning curve of C-IONM:**

Learning methods (multiple choices):

□The study class □Conference □Self-study

volume: □10 □50 □100 □＞100 Time (year): □1 □2 □＜5 □＞5

**3.Endoscopic thyroid surgery**

**Learning curve：**

Learning methods (multiple choices):

□The study class □Conference □Self-study

volume: □10 □50 □100 □＞100 Time (year): □1 □2 □＜5 □＞5

**Endoscopic surgery method (multiple choices)：**

□Transaxillary □BABA □Retroauricular □Facelift □Transcervical

□MIVAT □The transoral approach □Breast and breast approach

□Others

**What kind of intermittent monitoring stimulation device is used when I-IONM is applied during endoscopic surgery?**

□Percutaneous probe

□Lengthen the stimulus probe

□Surgical anatomical instruments modified probe

□Metal wire modified probe

**What kind of continuous monitoring stimulation device is used when C-IONM is applied during endoscopic surgery?**

□Percutaneous probe

□Lengthen the stimulus probe

□Surgical anatomical instruments modified probe

□Metal wire modified probe

□APS

**The reasons for applying IONM in endoscopic thyroid surgery (multiple choices)：**

□Locate nerve

□Identify nerve

□Evaluate neural function in real time during dissection

□Predict the vocal fold function

□Provide evidence-based evidence for neural function

□Avoid bilateral recurrent laryngeal nerve injury

□Monitor the superior laryngeal nerve

□Achieve continuous neurological monitoring

□Short operative time

□Increase the confidence of the surgeon

□Avoid the risk of medical disputes

□Have more advantages in complex thyroid surgery

□Teaching

□Scientific research

**The proportion of conversion from endoscopic surgery to open thyroid surgery:**

□＜1% □1-5% □5-10% □＞10%

**The proportion of conversion from robotic surgery to open thyroid surgery:**

□＜1% □1-5% □5-10% □＞10%

**4. Open thyroid surgery**

**Learning curve：**

Learning methods (multiple choices):

□The study class □Conference □Self-study

volume: □10 □50 □100 □＞100 Time (year): □1 □2 □＜5 □＞5

**The reasons for applying IONM in open thyroid surgery (multiple choices)：**

□Locate nerve

□Identify nerve

□Evaluate neural function in real time during dissection

□Predict the vocal fold function

□Provide evidence-based evidence for neural function

□Avoid bilateral recurrent laryngeal nerve injury

□Monitor the superior laryngeal nerve

□Achieve continuous neurological monitoring

□Short operative time

□Increase the confidence of the surgeon

□Avoid the risk of medical disputes

□Have more advantages in complex thyroid surgery

□Teaching

□Scientific research

**5. Application of IONM in thyroid surgery**

**Neuromonitoring system malfunction included (multiple choices):**

□The initial EMG signal is low

□EMG signal is lost during the operation

□The interference of monitoring signal is obvious

□It is difficult to detect the decrease of EMG in time

□Overdose of muscle relaxant

□Monitoring catheter failure

□Others

**Is there a regular anaesthetist:**

□Yes (□Trained □Untrained) □No

**6. Criteria for superior laryngeal nerve monitoring during thyroid surgery:**

□No routine monitoring

□Routine monitoring

□Determined by cricothyroid fibrillation

□EMG signal S1

□EMG signal S2

**7. The volume of IONM in your department in 2016 was approximately?**

□＜10

□10-50

□50-100

□100-500

□500-2000

□＞2000

**8. What kind of neuromonitoring systems are applied in your department?**

□Medtronic □Inomed □Dr. Langer □NCC □Other：

**9. CNMSG welcomes you to join us,**

If you wish, please fill out the confirmation again:

Name: Affiliations: E-mail: Tel:

**What are your suggestions for the future work of the CNMSG?**

**Chinese Neural Monitoring Study Group**


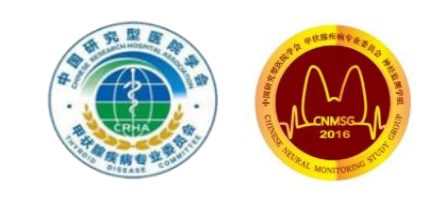

Supplement: Supplementary file 2 [file js9-111-0439-s002.docx]
