# Supplementary material for: Utilization of recurrent laryngeal nerve monitoring during thyroid surgery in China: a point prevalence survey (2015–2023)
Source: Int J Surg. 2024 Sep 6;111(1):439–49. doi: 10.1097/JS9.0000000000002084 (PMC11745604; doi:10.1097/JS9.0000000000002084)
Supplement: Supplementary file 3 [file js9-111-0439-s003.doc]

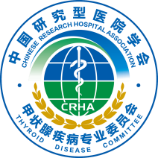

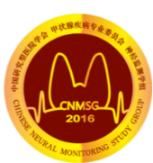
**Chinese Neural Monitoring Study Group（CNMSG）Questionnaire Survey**

Hello! Under the historical situation of the expansion of the training centers of intraoperative neural monitoring (IONM) in China and the further popularization of IONM technology, in order to better define the existing functions of training centers, communicate management experience, share academic achievements and exchange training experience, please fill out this questionnaire carefully. Thank you again for your active participation and look forward to your valuable suggestions to assist the study group in developing future work plans! (No substitution or false data)

Name: Gender: □man □woman Age: E-mail:

Affiliations: Department: Tel:

Professional title:□Chief physician □Associate chief physician

□Attending physician □Physician

IONM training centers establishment date:

Please complete the following table according to the data of thyroid surgery from

January 2017 to December 2017:

| **Thyroid surgery** | **Open surgery** | **Endoscopic surgery** | **Robotic surgery** |
| --- | --- | --- | --- |
| **Thyroid surgery volume** |  |  |  |
| **Proportion of complex thyroid surgery (%)** |  |  |  |
| **Proportion of thyroid cancers (%)** |  |  |  |
| **Tumor breakthrough the glands or suspicious lymph nodes in the central region (%)** |  |  |  |
| **Proportion of large masses (%) (weight > 100g or pressure on surrounding tissue)** |  |  |  |
| **Proportion of other complex thyroid surgery (%)**  **The reason:** |  |  |  |
| **Proportion of I-IONM (%)** |  |  |  |
| **Proportion of C-IONM (%)** |  |  |  |
| **IONM indicators (multiple choices) (%)** | □L1□V1  □R1□R2  □V2□L2 | □L1□V1  □R1□R2  □V2□L2 | □L1□V1  □R1□R2  □V2□L2 |
| **Proportion of monitoring failures (%)** |  |  |  |
| **Reasons for monitoring failures** | □  Muscle relaxant interference  □Monitoring catheter  □  Patient interface box  □Mainframe  □  Stimulus probe  □  Others: | □  Muscle relaxant interference  □Monitoring catheter  □  Patient interface box  □Mainframe  □  Stimulus probe  □  Others: | □  Muscle relaxant interference  □Monitoring catheter  □  Patient interface box  □Mainframe  □  Stimulus probe  □  Others: |
| **Solution** | □Resolved, method:    □Unsolved | □Resolved, method:    □Unsolved | □Resolved, method:    □Unsolved |
| **Proportion of recurrent laryngeal nerve (RLN) injury (%)** |  |  |  |
| **Proportion of RLN is dissected in detail (%)** |  |  |  |
| **Proportion of temporary injury (%)** |  |  |  |
| **Proportion of permanent injury (%)** |  |  |  |
| **Clear damage mechanism** | □Yes  □No | □Yes  □No | □Yes  □No |
| **Monitoring the superior laryngeal nerve (SLN)** | □Yes  □No | □Yes  □No | □Yes  □No |
| **Proportion of SLN injury (%)** |  |  |  |

IONM: Intraoperative neural monitoring

C-IONM: Continuous intraoperative neural monitoring

I-IONM: Intermittent intraoperative neural monitoring

**Part one: IONM training center management**

**1. The characteristics of your training center (multiple choices):**

□Standardized intraoperative neuromonitoring

□Application of IONM technology in complex surgery

□Application of C-IONM

□Application of IONM technology in endoscopic or robotic surgery

□Research related to animal experiments

□Others:

**2. The main training methods of your training center (multiple choices):**

□Theoretical study class

□Academic conference

□Surgical demonstration

□Animal experiment

□Others:

**3. The composition of the trainer in your training centers:**

The number of trainers with 100 or fewer IONM surgeries per year:

The number of trainers with 100-300 IONM surgeries per year:

The number of trainers with above 300 IONM surgeries per year:

The number of chief physicians:

The number of associate chief physicians:

The number of attending physicians:

The number of other professional titles:

**4. How can the trainers of your training centers obtain the IONM training qualification?**

□By training in other training centers (e.g. national training centers)

□By learning at domestic or international conferences

□By training at own training center

□By learning from experience in clinical practice

□By learning from experience in scientific research

□By self-study of literature or textbooks

□Others:

**5. The contents of theoretical lectures in your training center (multiple choices):**

□IONM technology basics principles

□IONM technology basic operation method

□IONM technology case share

□IONM technology latest research results sharing

□IONM technology expands applications

□Others:

**6. The number of IONM research publications in your training center:**

□Less than 3 □3-5 □6-10 □Above 10

Whether have published IONM SCI articles: □No □Yes, number:

**7. The number of domestic and international conferences attended by the trainers in your training center:**

Domestic conferences:

□Less than 3 per year □3-5 per year □6-10 per year □Above 10 per year

International conferences:

□Less than 3 per year □3-5 per year □6-10 per year □Above 10 per year

**8. The number of lectures by relevant experts from outside centers:**

□Less than 3 per year □3-5 per year □6-10 per year □Above 10 per year

Whether the International Neural Monitoring Study Group (INMSG) members are included: □No □Yes, name:

**Part two: IONM frontier results sharing**

**1. The application of C-IONM:**

□Applied in routine surgery

□Applied in special cases

□Applied in endoscopic or robotic surgery

□Applied in clinical research

□Applied in animal experiments

□Never applied

**2. The application methods of C-IONM (multiple choices):**

□Continuous vagus nerve monitoring with application of APS electrodes

□Other continuous monitoring fixed to the vagus nerve, such as:

□Surgical instrument probe forceps monitoring

□C-IONM with the aid of assistants

□Other methods:

**3. Improvements to the existing IONM equipment hardware:**

Stimulus side, such as stimulus probe and APS electrode, briefly describe your ideas for improvement:

Receiving side, such as monitoring catheter, briefly describe your ideas for improvement:

Recording side, such as host system and operation interface, briefly describe your ideas for improvement:

Other equipment, such as appearance, peripherals, interface box, briefly describe your ideas for improvement:

Addition of new hardware equipment to make the whole system more complete, briefly describe your ideas for improvement:

**4. The application of IONM in endoscopic or robotic surgery**

□Application of C-IONM mainly

□Application of I-IONM mainly

□Innovative devices or innovative methods of IONM have been applied, e.g., integrated probes for surgical instruments, extended probes, etc.

□Do not apply IONM in this type of surgery

**5. Which entry route in endoscopic thyroid surgery facilitates the application of IONM, (multiple choices):**

Entry routes favoring the application of the I-IONM

□Transaxillary □BABA □Retroauricular □Facelift

□Transcervical □MIVAT □The transoral approach

□Breast and breast approach

□Others:

Entry routes favoring the application of the C-IONM

□Transaxillary □BABA □Retroauricular □Facelift

□Transcervical □MIVAT □The transoral approach

□Breast and breast approach

□Others:

**6. The reasons for applying IONM in endoscopic or robotic surgery, (multiple choices):**

□Locate nerve

□Identify nerve

□Evaluate neural function in real time during dissection

□Predict the vocal fold function

□Provide evidence-based evidence for neural function

□Avoid bilateral recurrent laryngeal nerve injury

□Monitor the superior laryngeal nerve

□Achieve continuous neurological monitoring

□Short operative time

□Increase the confidence of the surgeon

□Avoid the risk of medical disputes

□Have more advantages in complex thyroid surgery

□Teaching

□Scientific research

**7. The reasons for applying IONM in open surgery, (multiple choices):**

□Locate nerve

□Identify nerve

□Evaluate neural function in real time during dissection

□Predict the vocal fold function

□Provide evidence-based evidence for neural function

□Avoid bilateral recurrent laryngeal nerve injury

□Monitor the superior laryngeal nerve

□Achieve continuous neurological monitoring

□Short operative time

□Increase the confidence of the surgeon

□Avoid the risk of medical disputes

□Have more advantages in complex thyroid surgery

□Teaching

□Scientific research

**8. If there is a failure of IONM, such as signal interference affecting monitoring, unexplained loss of signal, low initial signal, etc., what countermeasures could be taken, (multiple choices):**

□Be able to apply relevant theories to explain the causes of failure

□Be able to analyze causes and take remedial action in a timely manner

□Other specialists in own center can help resolve the issue in a timely manner

□Ask for remote help from specialists in other centers

□Although it was not solved, it can be summarized based on the analysis of the causes to avoid reoccurrence

□Summarizing the lessons and increasing relevant professional knowledge

□Abandonment of the application of IONM for the time being

**Part three：IONM basic medical institution training experience**

**1. The number of basic medical institutions radiated from your training center:**

□Less than 3 □3-5 □6-10 □Above 10

**2. Whether to radiate basic medical institution across provinces and regions:**

□No □Yes, the number and name of specific medical institutions:

**3. Department composition of trainees, (multiple choices):**

□General surgery □Otolaryngology - head and neck surgery

□Thyroid Surgery □Anesthesiology □Other department

**4. Professional title composition of trainees:**

Chief physician % Associate chief physician %

Attending physician % Physician %

**5. When would trainees apply the IONM, (multiple choices)?**

□Routine application

□Applied to malignant cases

□Applied to large benign masses

□Assessment of surgical difficulty by preoperative ultrasound, with application to complex malignant cases

□It can be applied in the cases of abnormal vocal cord movement indicated by preoperative laryngoscopy

**6. Investigation on the application of IONM equipment**

□IONM equipment was purchased prior to attending the training, but was not applied correctly and the application benefits were low

□IONM equipment was purchased prior to attending the training and was able to perform basic operations, but the application was not standardized and not effective

□Idea of purchasing IONM equipment after attending a training session

□Idea of purchasing IONM equipment after attending a training session, but was constrained by factors such as health insurance or the cost of the equipment

□Intended to abandon the application of IONM after attending a training session

**7. After IONM training, participants can learn, (multiple choices):**

□IONM basic operations

□IONM fault diagnosis and troubleshooting

□IONM system setup (including anesthesia, setup, etc.)

□IONM surgery case sharing

□IONM in endoscopic and robotic surgery

□IONM clinical research

□IONM animal experimental research

□C-IONM technology

□Innovative equipment and technology applications

□The economic benefits of IONM

□The scientific value of IONM

□Hospital overhead costs for IONM

**8. What kind of format course organization do you think is appropriate for your training center?**

□Theoretical lectures only

□Demonstration in the operating room

□Animal experiment demonstration

□Learning in clinical practice

□Personal guidance by trainers

**Chinese Neural Monitoring Study Group**

**
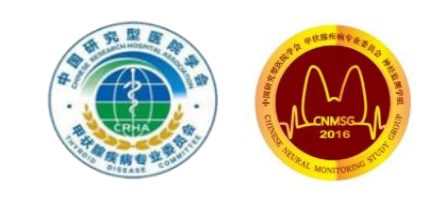
**
