# Supplementary material for: Utilization of recurrent laryngeal nerve monitoring during thyroid surgery in China: a point prevalence survey (2015–2023)
Source: Int J Surg. 2024 Sep 6;111(1):439–49. doi: 10.1097/JS9.0000000000002084 (PMC11745604; doi:10.1097/JS9.0000000000002084)

**
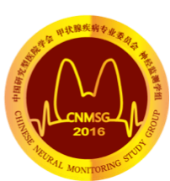

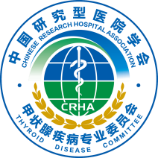
Chinese Neural Monitoring Study Group Questionnaire Survey（2023.04）**

Hello! Intraoperative neural monitoring (IONM) has been carried out in thyroid surgery for many years in China. In order to understand the current status of clinical application and training demands of IONM technology, and to promote the sustainable development of IONM technology in China, the Chinese Neural Monitoring Study Group (CNMSG) conducted this questionnaire survey. Looking forward to your suggestions and thank you for your active participation!

Name: Professional title: Affiliations: Department:

**Part one: IONM technology application and popularization**

Please faithfully fill in the following relevant data of your affiliations.

1. **Average annual number of thyroid-related surgeries:**

Average annual number: The proportion of thyroid malignant tumor surgery:

1. **When to start applying IONM technology?**

In

1. **How many surgeries were performed with the help of IONM**

Average annual number: Cumulative number:

1. **What is the current proportion of thyroid-related surgeries using IONM technology?**

□Routine（Above 90%） □Often (Above 60%)

□Sometimes (Above 30%) □Seldom (Below 10%)

1. **What is the annual incidence of postoperative hoarseness?**

□Below 1% □1%~3% □3%~5% □5%~10% □Above10%

1. **What are the main reasons for choosing to apply IONM？**

□Assisting in identifying dissociated nerves

□Reducing the risk of nerve damage

□Shortening the duration of surgery

□Shortening the learning curve of young doctors

□Medical litigation needs

□Others:

1. **What are the main reasons for limiting to apply IONM?**

□This technology has not yet been introduced

□Lack of anesthesiology cooperation

□The clinicians are inexperienced

□Cost problem

□Preferences of the patient

□No limits

□Others:

1. **What are the indications for IONM？**

□Routine application

□Complicated and difficult operation

□Endoscopic thyroid surgery

□Request of the patient

□Others:

1. **What is the hardware for applying IONM？（If there are multiple choices, please fill in the proportion of usage）**

□Monitor endotracheal tube

□Needle recording electrodes

□Continuous intraoperative neural monitoring

□Probe forceps

□Others:

1. **What types of IONM instruments are used？（Please fill in the quantity in parentheses）**

□Medtronic NIM 2.0（ ） □Medtronic NIM 3.0（ ） □NCC XP-1E（ ）

□Inomed-C2（ ） □Other：

**Part two: IONM theoretical knowledge and professional skills**

1. **Whether the vocal cord function is examined during the perioperative period?**

□Routine preoperative and postoperative checkups

□Routine preoperative checkups, postoperative checkups as needed

□Routine preoperative checks, no postoperative checkups

□Preoperative and postoperative checkups as needed

□No preoperative and postoperative checkups

1. **What methods are used to perform vocal cord motor function tests?**

□Electronic laryngoscope □Stroboscopic laryngoscope

□Laryngeal ultrasound □Noise acoustic analysis □Other：

1. **Is the vagal V1 signal routinely monitored before the surgical field operation?**

□Surgical side only □Bilateral sides □Depending on the circumstances

1. **Is the vagal V2 signal routinely monitored after the surgical field procedure?**

□Surgical side only □Bilateral sides □Depending on the circumstances

1. **Is monitoring of the external branch of the superior laryngeal nerve performed?**

□Routine monitoring

□Monitoring during high-risk surgery

□Monitoring only in relation to the upper polar region

□Do not monitor

1. **What is the average initial amplitude of V1 signal in general?**

□About 500µV □500µV~1000µV □Above 1000µV

1. **What is the rate of intraoperative electromyographic abnormalities?**

□1%~5%　□5%~10%　□10%~15%　□15%~20%　□Above 20%

1. **Is it known what to do if an abnormal electromyographic signal occurs intraoperatively?**

□Complete mastery □Partial understanding □Don't understand

1. **What is the rate of intraoperative electromyographic signal loss (<100μV)?**

□Below 1% □1%~3% □3%~5% □5%~10% □Above 10%

**Part three: IONM** **training and learning**

1. **Have you ever participated in IONM related training courses or workshops?**

□Participated in regional offline courses

□Participated in the national advanced training courses

□Only heard relevant lectures at academic conferences

□Never participated in systematic training

1. **The number of thyroid surgical team members in your institution** ；

**And the number of personnel who attended training courses**

1. **Through what channels did you learn about IONM related technical training courses?**

□WeChat or public number push □Peer introduction □No way to know

1. **Which aspect of the training content would you like to learn more about?**

□IONM principle, parameters and equipment

□IONM system establishment and setup

□Key points of IONM operation

□Analysis and treatment of abnormal electromyography signals

□Methods and directions for carrying out scientific research

1. **Which of the following training methods do you prefer?**

□Offline small group teaching lectures

□Training in live surgical demonstrations

□Online replayable video courses

□Comprehensive training mode

1. **Which of the following training materials do you prefer?**

□Printed materials designed for each training courses

□Interpretation of authoritative guidelines

□Standardized practical demonstration video

□Video compilation of surgical cases

1. **What channels do you usually learn about the progress of IONM related fields?**

□Journal monographs □Public number □Academic conferences □Others

1. **Does your institution conduct scientific research related to IONM?**

□Yes □No

1. **Does your institution have published any academic papers related to IONM?**

□Yes（SCI: ; [Core journals](https://cn.bing.com/dict/search?q=core%20journals&FORM=BDVSP2&qpvt=chinese+core+journals) ;） □No

1. **How many training activities does your organization hold each year?**

□Never held □Below 4 □4~12 □Above12

1. **How many people have participated in the training activities held by your institution？**

□Below 50 □50~100 □100~300 □300~1000 □Above1000

1. **How well do you know the Chinese Guidelines on IONM 2023 Edition？**

□Not yet understood

□Known to have been released but not yet read in detail

□Read the whole article

1. **What form of supporting interpretation do you want to introduce in Chinese Guidelines on IONM 2023 Edition?**

□Interpretation by experts

□Simplified version

□Detailed version

□Video speech

□Presentation guidelines

□Others

**Part four: Chinese Neural Monitoring Study Group and** **IONM training centers**

**1. What aspects of arrangement would you like the Chinese Neural Monitoring Study Group to improve?**

□Carry out more regular training courses

□Carry out more advanced training courses

□Carry out more teacher training courses

□Publish more readable textbooks or monographs

□Establish an academic community for communication and inquiry

□Establish an official account, and timely push academic progress and relevant class or conference information

□To supplement the regional training centers and the committee members

□Others

**2. Please express your views on the work of the Chinese Neural Monitoring Study Group.**

**Chinese Neural Monitoring Study Group**


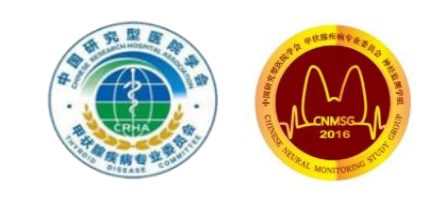

Supplement: Supplementary file 4 [file js9-111-0439-s004.docx]
